# Single molecule mass photometry reveals the dynamic oligomerization of human and plant peroxiredoxins

**DOI:** 10.1016/j.isci.2021.103258

**Published:** 2021-10-13

**Authors:** Michael Liebthal, Manish Singh Kushwah, Philipp Kukura, Karl-Josef Dietz

**Affiliations:** 1 Department of Biochemistry and Physiology of Plants, Faculty of Biology, University of Bielefeld, 33615 Bielefeld, Germany; 2 Physical and Theoretical Chemistry Laboratory, Department of Chemistry, University of Oxford, South Parks Road, OX1 3QZ Oxford, UK; 3 The Kavli Institute for Nanoscience Discovery, Oxford, UK

**Keywords:** Biophysical chemistry, Protein, Structural biology

## Abstract

Protein oligomerization is central to biological function and regulation, yet its experimental quantification and measurement of dynamic transitions in solution remain challenging. Here, we show that single molecule mass photometry quantifies affinity and polydispersity of heterogeneous protein complexes in solution. We demonstrate these capabilities by studying the functionally relevant oligomeric equilibria of 2-cysteine peroxiredoxins (2CPs). Comparison of the polydispersity of plant and human 2CPs as a function of concentration and redox state revealed features conserved among all 2CPs. In addition, we also find species-specific differences in oligomeric transitions, the occurrence of intermediates and the formation of high molecular weight complexes, which are associated with chaperone activity or act as a storage pool for more efficient dimers outlining the functional differentiation of human 2CPs. Our results point to a diversified functionality of oligomerization for 2CPs and illustrate the power of mass photometry for characterizing heterogeneous oligomeric protein distributions in near native conditions.

## Highlights

• Mass photometry resolves oligomerization dynamics of 2-Cys peroxiredoxins
• The thiol redox transition can be tuned by mutagenesis of amino acid residues
• Plant and human 2-Cys peroxiredoxins have common and unique features

## Graphical abstract

**Figure:**
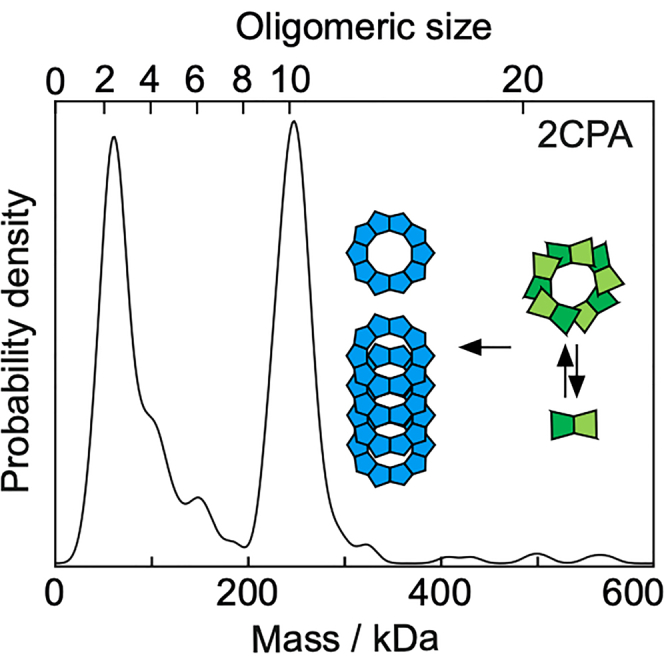

## Introduction

Protein oligomerization – crucial for biological function and regulation of proteins – is driven by both homotypic and heterotypic protein interactions. Oligomerization is a broadly used term referring to a wide range of processes, such as dimerization and tetramerization, ring-like structure formation, macroscopic filament formation, or numerous intermediates of a functional protein assembly. Oligomerization of protein complexes consisting of multiple subunits also brings numerous advantages such as stability to denaturation, error control and regulation of function without the need for protein turnover (Goodsell and Olson, 2000). Experimental characterization of the inevitable mixtures of oligomeric states for various protein systems, and thus the formation and disassembly of functional states; however, is challenging. The underlying reason is the heterogeneity of species present in solution, and the resulting requirements in terms of resolution, sensitivity and dynamic range for any technique aimed at characterizing that heterogeneity.

2-Cysteine peroxiredoxins (2CPs) are the largest subgroup in the family of peroxiredoxins (PRX) and are a prototypical example of the range and functional importance of oligomerization. They fulfill diverse functions across the kingdoms of life and within the same cell, depending on their degree of oligomerization, which in turn is controlled by their redox state (Wood et al., 2002; Dietz, 2011; Poole and Nelson, 2016). Initially, mainly considered as thiol peroxidases to scavenge reactive oxygen species (ROS) under regular and stress conditions, their role in various cellular mechanisms with redox-dependent processes ranges from cell differentiation, tumor suppression, signal transduction, thioredoxin (TRX) oxidation and chaperone-dependent protein homeostasis to participation in stress resistance and disease control (Neumann et al., 2003; Jang et al., 2004; Hall et al., 2009; Yan et al., 2009; Vaseghi et al., 2018).

**Figure 1:**
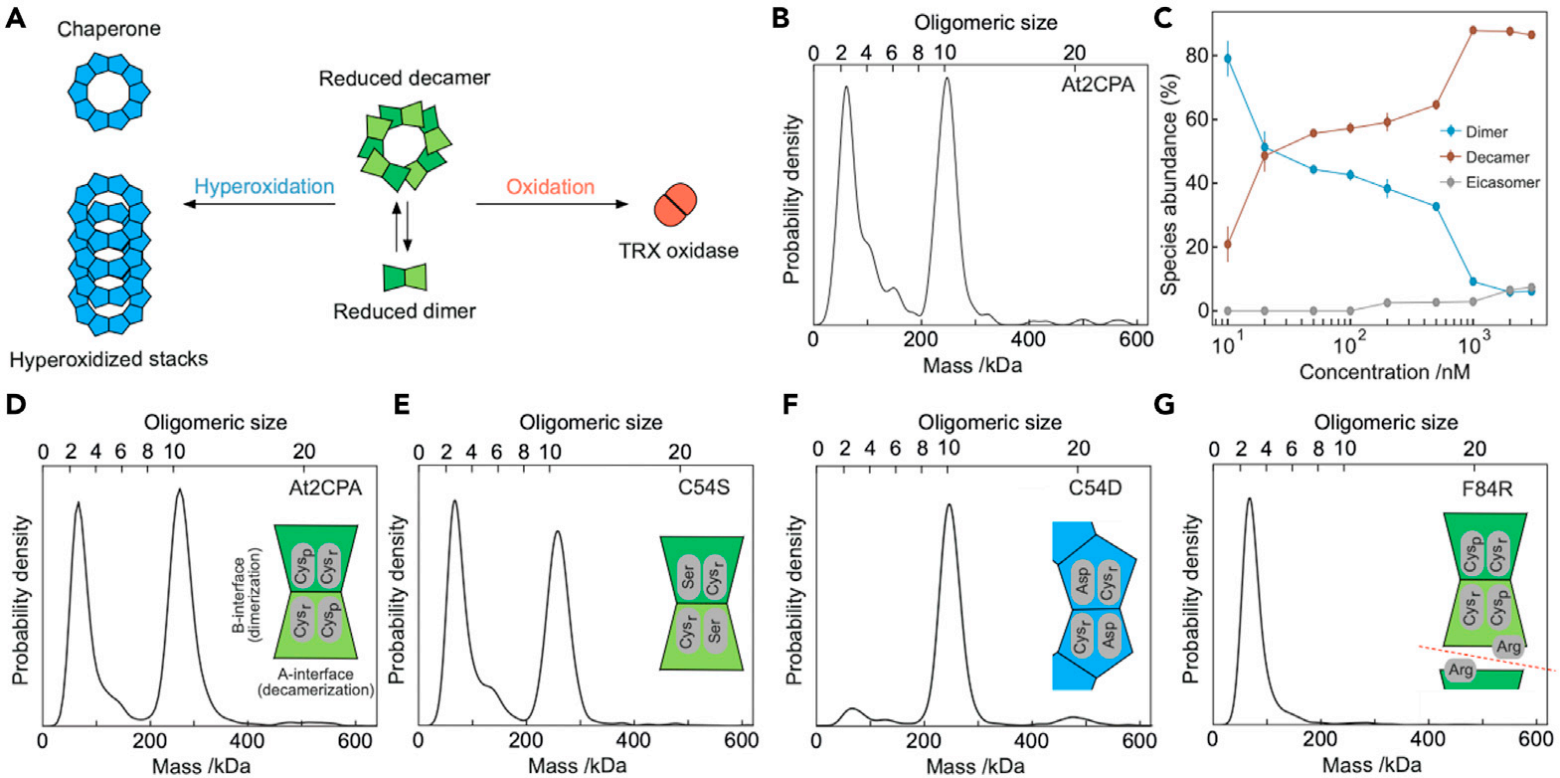
Mass photometry of reduced *Arabidopsis* 2CPA (At2CPA) and site-directed mutated variants (A) Schematic of known oligomeric species of At2CPA, their oxidation state-dependent interconversion and function. (B) Mass distribution for 20 nM At2CPA. Note that detection events are counted corresponding to molecules e.g, one count dimer contains two monomers, one count decamer ten monomers. Also, see Figure S1. (C) Relative abundance of specific oligomer structures of At2CPA as a function of total protein concentration. Data represent means ± standard deviation (SD). (D–G) Conformational state of site-directed mutated variants of At2CPA at 100 nM concentration. The schematics illustrate the mutations introduced. Statistics: All experiments were performed with *n* > 6 determinations on at least two different days and with two different protein preparations (see Figure S2).

Their polydispersity-dependent functions range from chaperone activity (hyperoxidized decamer, including high molecular weight aggregates (HMW), a term usually used for assemblies larger than decamers, acting as a peroxidase (mixture of reduced dimers and decamers), redox signaling element (oxidized dimer) to conformation-specific protein-protein interactor (Figure 1A) (Cao and Lindsay, 2017). Other types of PRX exhibit little oligomerization propensity and predominantly persist as monomers or dimers (PRXQ/bacterioferritin comigratory protein BCP) or adopt different quaternary structures (PRX type II) (Dietz, 2011). Oligomerization thus controls protein activity, protein turnover and exposure of interaction surfaces (Ali and Imperiali, 2005). The complexity of oligomerization in relation to function increases further in fungi, mammals, and humans, where multiple 2CP isoforms have been identified (Wong et al., 2002; Nelson et al., 2011). The dynamic oligomerization behavior of these proteins is far from understood. Additionally, they localize to different subcellular compartments, e.g., in human cells either to cytosol (HsPRX1, HsPRX2) or to mitochondria (HsPRX3) indicating distinct and location-specific function in metabolism and regulation of cell processes. The difficulties to accurately measure oligomeric distributions and dynamic transitions with current techniques complicate the ability to faithfully correlate protein oligomerization with PRX function and localization.

Reported oligomeric states for 2CPs are dominated by dimers, decamers and large aggregates and have been characterized by a variety of bioanalytical techniques (Gourlay et al., 2003; Meissner et al., 2007; Barranco-Medina et al., 2008; Cao et al., 2015; Liebthal et al., 2016; Morais et al., 2017). For the majority of solution-based techniques, however, resolving different oligomeric states in a mixture, as expected in thermodynamic equilibrium, is not possible, or requires prior chromatographic separation. As a result, while these studies have shown beyond doubt that 2CP is polydisperse and that changes in oligomeric state can be traced to function, our understanding of the molecular details of PRX polydispersity beyond fairly general observations in steady states, and its dependence on redox conditions, dynamic changes to the oligomerization interfaces and ultimately function, remains limited.

Some of the current key questions include (i) the precise distribution profile of the dimeric and oligomeric states and its dependence on structural features, (ii) the nature of transitions between oligomeric states as a result of a dynamic physicochemical environment, (iii) the kinetics of conformational transitions depending on signaling cues like changing redox conditions and (iv) the effect of interacting partners. Given the polydispersity of 2CP, we reasoned that mass photometry as a method for label-free detection and mass measurement of individual proteins and their assemblies in solution (Young et al., 2018) could be ideally suited to provide quantitative information on the interplay between different oligomeric states, and how they depend on redox state, the molecular details of the oligomerization interface and the species origin. The critical performance parameters of mass photometry concern high dynamic range detection as a result of single molecule operation, unmatched mass resolution in the context of solution-based technologies, and the speed and ease of operation enabling screening of different constructs under a range of environmental conditions.

## Results

### Concentration-dependent oligomerization of 2CP

First, we tested the suitability of mass photometry to scrutinize the oligomerization state of 2CP. Reduced Arabidopsis 2CPA (At2CPA), one of the two isoforms encoded in the genome of *Arabidopsis thaliana*, at 20 nM exhibited an oligomeric mixture dominated by dimers (48 kDa) and decamers (240 kDa), with tetramers, and hexamers appearing as distinct peaks with exponentially decreasing abundance (Figure 1B). Equal abundance of dimeric and decameric oligomers indicates five times more dimer being associated with the decameric fraction under these conditions (Figure S1). HMW aggregates (>240 kDa) exhibited very low abundance, with the overall distribution being highly reproducible with identical and different protein preparations (Figure S2). Given that the detection limit of mass photometry is on the order of 40 kDa, we could not detect monomeric species, which is unlikely to be limiting in this case, given that dimers have consistently been reported as the minimal building block of 2CPs in the literature (König et al., 2013, and reviewed in Dietz, 2011). In addition, owing to the proximity of the dimer mass to the detection limit of our instrument, the dimer peak frequently appeared at slightly higher than expected mass.

We then investigated the dependence of the dimer-decamer equilibrium on bulk protein concentration (Figure 1C). Although the concentration dependence did not enable a simple determination of the associated dissociation constant, it suggests a *K*_*d*_ for dimer and decamer species on the order of a few 10 nM, in agreement with the distribution in Figure 1B. The onset of eicosamer formation at 2 μM total protein concentration suggests their formation also under physiological conditions in cells (30–60 μM, depending on organism) but upper concentration limits of mass photometry did not enable further quantifications above 3 μM in this case.

Given the importance of decamer stability for the formation of HMW, we decided to further investigate the dimer-decamer equilibrium in protein variants obtained by site-specific mutagenesis of amino acid residues in the catalytic center and at the oligomerization interfaces. We chose amino acid substitutions to mimic specific conformations and to address the redox dependence of the oligomer/dimer-equilibria (König et al., 2013). Specifically, we stabilized the reduced conformation by mutating the peroxidatic C54 to serine (C54S) preventing disulfide formation, which resulted in a small decrease in decamer abundance compared to the wildtype (Figures 1D and 1E), further confirming that the dimer is the minimal unit and dimer formation is insensitive to redox conditions. Substituting Asp for C54 (C54D) mimics the hyperoxidized and charged sulfinyl group and stabilized the decamers and larger aggregates with known chaperone function (Figure 1F). The dimer fraction was extremely small (Wood et al., 2002; Dietz, 2011).

Conversely, introducing a charged arginyl residue at the dimer-dimer interface in the F84R variant inhibited decamer formation completely, causing almost exclusive accumulation of dimers and minimal abundance of tetramers (Figure 1G). We confirmed the mass photometry results using dynamic light scattering at higher protein concentrations of 10 μM, albeit at much lower mass resolution (Figure S3). The oligomerization state thus sensitively responded to introduced changes in amino acid side chains suggesting a delicate interplay between oligomerization and structural features (König et al., 2003).

**Figure 2:**
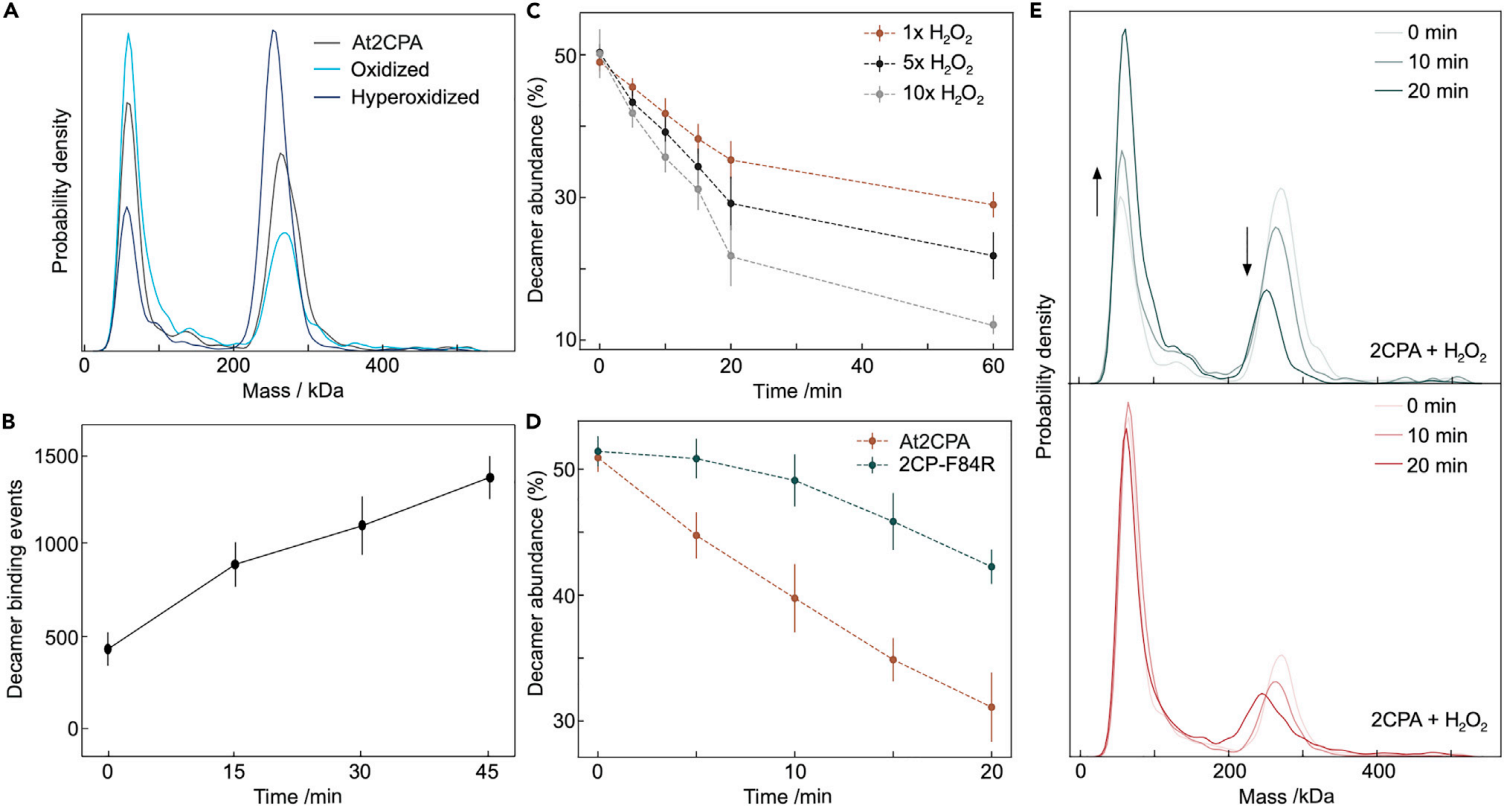
Thiol-oxidation induced changes in oligomerization state of At2CPA (A) Mass distributions of reduced (black line), oxidized (light blue) and hyperoxidized (dark blue) At2CPA at 100 nM concentration. Oxidation was achieved by incubating reduced At2CPA with 1 μM H_2_O_2_ for 30 min. Hyperoxidation was induced by continuous peroxidase turnover of At2CPA by incubation with equal amounts of H_2_O_2_ and DTT (both 1 μM). (B) Generation of reduced At2CPA decamers (mean ± standard deviation (SD)) from oxidized dimers and regeneration by reduced thioredoxin (TRX-x) in 5-fold excess (5 μM) as a function of time. (C) Decamer abundance (mean ± SD) as a function of time-dependent oxidation of At2CPA by H_2_O_2_. H_2_O_2_ was added to 100 nM At2CPA at equimolar (1×) or excess (5× or 10×) molar amounts. (D) Protection of reduced At2CPA decamer abundance (mean ± SD) from oxidation by reduced F84R. A mix of WT and F84R at a ratio 1:1 was exposed to 5-fold H_2_O_2_. The control assay contained twice the WT amount. (E) Relative appearance of dimer and decamer in MP distributions including At2CPA (upper diagram) and a mix of At2CPA and F84R. Statistics: All experiments were performed with n > 6 independent determinations on at least two different days and using at least two different protein preparations. Relative and absolute decamer abundance was taken from single mass readings between 200 and 300 kDa.

Based on these results, we next decided to investigate how the redox state of At2CPA affects oligomerization through the peroxidatic cysteine (CysP54) reacting with H_2_O_2_ and forming a disulfide bond with the resolving cysteine (CysR176). CysP together with vicinal amino acid residues Arg, Thr, and Pro forms a catalytic pocket in the fully folded (FF) structure (Karplus and Hall, 2007). This molecular environment lowers the pKa of CysP below 6 under physiological conditions giving rise to an extraordinarily high reactivity toward peroxide substrates (Perkins et al., 2014). Upon oxidation, the protein conformation changes to the locally unfolded (LU) state allowing for disulfide formation with CysR, which exhibits a weakened dimer-dimer interface causing oligomer dissociation (Wood et al., 2002). In line with this model, we found that exposing 2CP to H_2_O_2_ caused a significant shift away from decamers toward dimers (Figure 2A). By contrast, hyperoxidation of the C54 thiol to its sulfinic acid derivative by simultaneous pre-incubation with H_2_O_2_ and dithiothreitol (DTT), which promotes repeated catalytic turnover and stimulates accumulation of sulfinic acid (König et al., 2013), led to an increase in the decamer-to-dimer ratio beyond that observed in the reduced state (Figure 2A).

Reductive regeneration of oxidized PRX in the cell is achieved by TRX or other electron donors (Liebthal et al., 2018). Accordingly, adding excess reduced TRX-x from Arabidopsis to oxidized 2CP caused re-association of reduced decamers over time (Figure 2B). We could not use the dimer-decamer ratio in this case, because all tested TRXs generated protein-like signatures in the 40–60 kDa range (Figure S4), and this readout for TRXs overlapped with the 2CP dimer, impeding evaluation of 2CP dimers. Nevertheless, by monitoring the presence of higher oligomers specific for 2CP, we could monitor redox transitions by counting single molecule populations even at very low concentrations.

These results demonstrate our ability to rapidly determine the dependence of the oligomeric distribution of 2CP on redox conditions. We therefore set out to quantify time- and concentration-dependent oxidation in the presence of 1×, 5× and 10× molar excess of H_2_O_2_ (Figure 2C). In line with results from Figure 2A, we found that H_2_O_2_-dependent oxidation increased the dimer population at the expense of decameric species (Figure 2C). The initial (0–20 min) dissociation of decamers accelerated with increasing H_2_O_2_ levels, while being largely independent of H_2_O_2_ abundance for later time points (20–60 min). Close inspection of the time-dependence of decamer decay revealed two-fold stimulated rates of disassembly at 10-fold increased H_2_O_2_ concentration. Apparently, oxidation to the sulfinic acid and disulfide form is more rapid than disassembly of the decamer into dimers. The kinetic constraints likely have implications for the structural and functional transitions and interactions with biological partners.

Given that oligomers larger than dimers have been observed *in vivo*, we next explored the relative efficiency of dimers vs. decamers in H_2_O_2_ detoxification (Seidel et al., 2010). To do this, we used the F84R variant, which exclusively exists as a dimer (Figure 1D) and combined it at a 1:1 molar ratio with WT decamers, before adding a 5-fold excess of H_2_O_2_. The amount of WT decamer served as a convenient readout of excess H_2_O_2_. In the presence of F84R, we observed almost complete protection of the wildtype decamer from oxidative destabilization during the first 5 min, with the protective effect compared to a sample lacking F84R prevailing until the end of analysis (Figure 2D). The corresponding histograms highlight the dissociation of decamers over time with a concomitant increase in dimers (Figure 2E). This increase was altered upon F84R addition due to the higher relative abundance of dimers compared to decamers and was considered in the calculations. These results suggest that the dimer is more readily involved in the peroxide reduction reaction, i.e. the more active form involved in the redox reactions, whereas the decamer may preferentially serve as a reserve pool of active dimers and in cell signaling.

**Figure 3:**
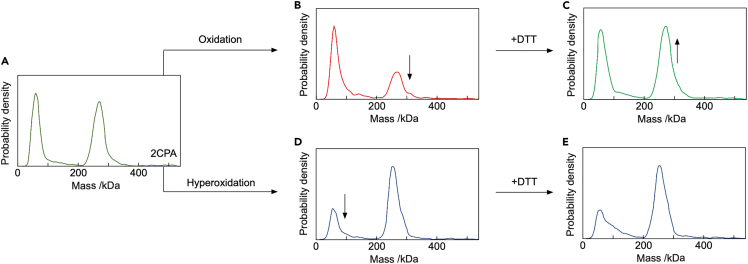
Mass distribution of At2CPA at 100 nM as affected by (hyper)oxidation and subsequent reduction (A–C) (A) Mass distribution of reduced At2CPA at 100 nM concentration, (B) after oxidation with H_2_O_2_, and (C) after reduction of the oxidized disulfide form by addition of 10-fold excess DTT. (D) Effect of hyperoxidation on dimer-to-decamer ratio. Hyperoxidation was achieved by addition of 10-times excess DTT and H_2_O_2_ in equimolar amounts. (E) The hyperoxidized sulfinyl form was insensitive to addition of a 10-fold excess DTT. All protein samples were treated with redox additives in excess amounts 30 min before dilution to 100 nM. Each sample was kept on ice for 20 min to ensure reproducibility. Statistics: Data are averaged values from *n* > 6 independent determinations performed at least on two different days and with two different protein preparations.

To further understand the effect of cellular redox state on the dynamics of 2CP oligomerization, we tested conditions that can restore decamer abundance under *in vitro* conditions. Reduced 2CP adopted the known equal distribution of dimer and decamer (Figure 3A). Single oxidation by H_2_O_2_ generated oxidized dimers (Figure 3B). DTT efficiently reduced the oxidized dimer and fostered accumulation of reduced decamers (Figure 3C). However, DTT failed to mediate the reversal for the hyperoxidized sulfinyl form of wildtype (Figures 3D and 3E). The hyperoxidized form accumulated as decamer and decameric stacks similar to the C54D variant which mimics exactly this state (Figure 1D). Hyperoxidation is enhanced under abiotic stress like heat resulting in exclusive accumulation of decamers and hyperaggregates likely by a differential charge distribution on the protein surface (Jang et al., 2004).

**Figure 4:**
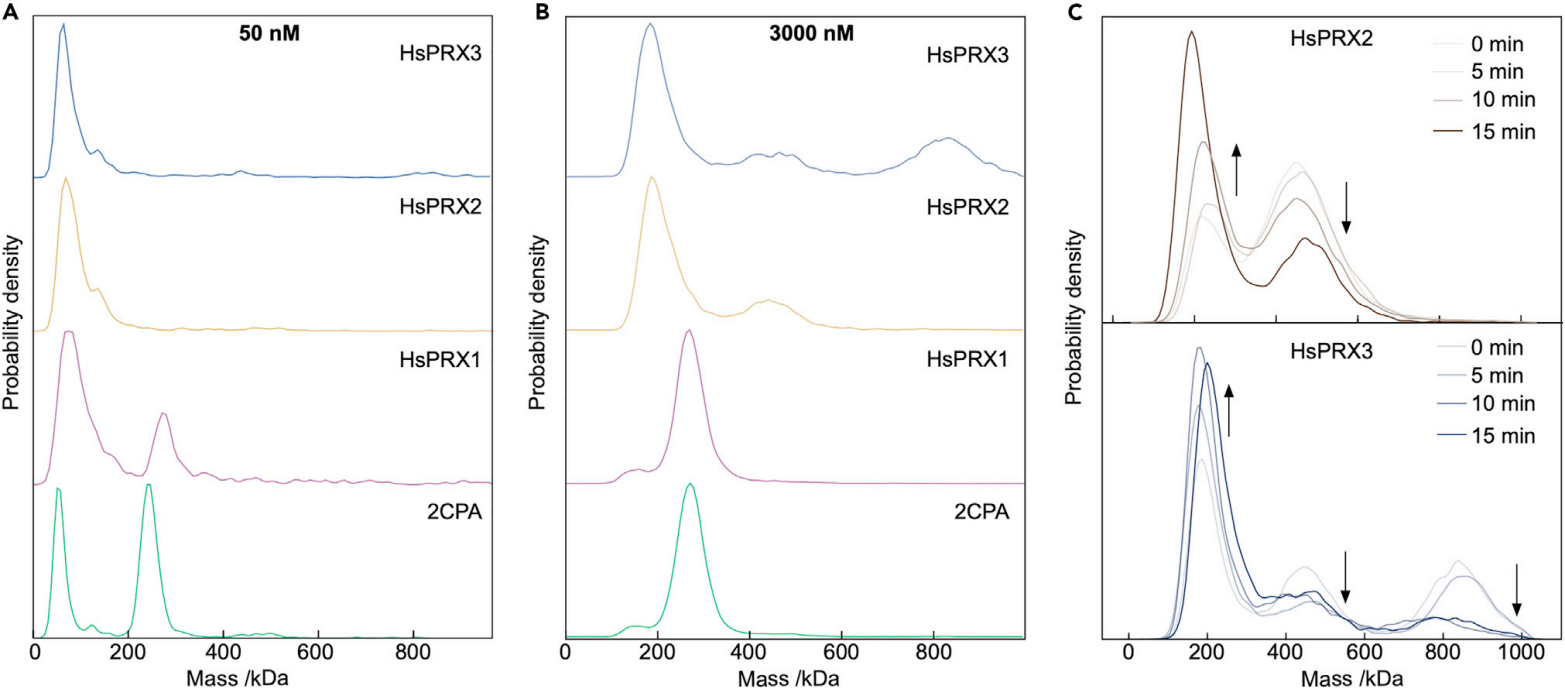
Diversification of oligomerization state of plant and human 2CPs (A) Oligomerization distribution of reduced human peroxiredoxins HsPRX1 (cytosol, pink), HsPRX2 (cytosol, orange), and HsPRX3 (mitochondria, blue) and plant 2CPA (chloroplast, green) at 100 nM total protein concentration. (B) Distributions at 3 μM total protein concentration, other details as in (A). (C) Effect of oxidation on the oligomerization of human 2CPs. Statistics: Data are averaged values from n > 6 independent determinations performed on at least two different days using at least two different protein preparations.

These observations of oligomer-specific activity and their dependence on redox conditions suggest that differences in oligomeric distributions may also be connected to function in different human 2CP isoforms. We therefore studied three human 2CPs encoded by the human genome. At 50 nM monomer concentration, HsPRX1 exhibited a similar oligomeric distribution as plant 2CP and did not adopt the eicosameric conformation (Figures 4A and S5). For HsPRX2 and HsPRX3 we could not find evidence for decamers, reminiscent of F84R (Figure 1G).

At 3 μM monomer concentration, HsPRX2/3 exhibited oligomer formation but compared to 50 nM smaller multimers like tetra-octamers, hexa-octamers and octamers were formed as well (but not distinguishable) with evidence for decamer stacking (Figure 4B). At 3μM HsPRX3 concentration, a stack of four decamers dominated with eicosamers present as well (Figure 4C). Similarly, we found signatures of eicosamers for HsPRX2, but little for HsPRX1. Taking monomer equivalents into stoichiometric ratios, we observed significant amounts of 2CP monomers stored in higher oligomeric structures for HsPrx2 and HsPrx3 (Figure S6). Oxidation by a 5-fold excess of H_2_O_2_ destabilized the decamer stacks resulting in an accumulation of smaller multimers, presumably dimers for both HsPRX2 and HsPRX3 (Figure 4C). Oxidation of eicosamer and eicosamer stacks of HsPRX3 resulted in an unequal decay for both oligomers favoring the largest HMW stack for oxidation.

## Discussion

Our results demonstrate the potential of MP for studying functionally relevant transitions, energetics, and dynamics in proteins where function is directly connected to specific oligomeric states. 2CP adopts five redox-dependent conformations namely reduced dimers, reduced decamers, oxidized dimers, hyperoxidized decamers and hyperoxidized stacks (Figure 1A) (Dietz, 2011; König et al., 2013). We find that the dimer-decamer equilibrium is already present in the low nM range, in contrast to previous studies using ITC and DLS reporting 2.1 μM for 2CP and 1.3 μM for human PRX1 (Barranco-Medina et al., 2008; Liebthal et al., 2016). Given the observation of decamer stacking at μM concentrations, these previous observations could possibly be assigned to the hyperaggregate (dis)assembly, though the cooperativity of 130 previously observed with ITC remains unexplained. It is possible that several processes contribute to this phenomenon, namely aggregate dissociation followed by decamer dissociation.

Our results confirm the presence of transient (intermediate) oligomeric states such as tetramers and hexamers and also assemblies between decamers and subdecamers e.g., decamer and hexamer. However, quantitative information on the stability of these intermediate states is unavailable. The varying oligomerization propensity of mutated 2CP variants demonstrates the sensitivity of the oligomerization process to molecular features such as introduced charges in the catalytic center (C54D) or at the dimer-dimer interface in F84R. The dimer-only F84R variant exhibits a 2.6-fold higher rate constant for H_2_O_2_-reduction than 2CP wild type (König et al., 2013). Our experiments provide the molecular basis for this observation. F84R exclusively remains dimer in solution with a highly active intact catalytic site. Presence of reduced F84R led to the reduced dissociation of the WT 2CP decamer, indicating that the dimer is the more active form involved in the redox reaction, and decamer dissociation follows depletion of the active reduced dimer (Figure 2D). The results demonstrate distinct roles of dimers and decamers, where dimers serve as the functionally active form and decamers serve as the storage form for releasing active dimer and likely as an interaction platform.

This result pinpoints to the functional significance of decamer formation as a selective force in evolution because improved thiol peroxidase performance of the constitutive dimer could have evolved by suppressing the ability for decamer formation for the plant 2CP. This may be different for HsPRX2 and HsPRX3 that were reported to display higher thiol peroxidase activity as decamers by stabilization of the active site loop-helix (Poole, 2008). In plants, the different peroxidase efficiency of dimers and decamers of At2CPA may avoid its complete oxidation and limit futile reduction-oxidation cycles in the cell.

The plant 2CP is reduced by TRX (König et al., 2003). This mechanism generates oxidized TRX which in turn oxidizes photosynthetic enzymes e.g., upon lowering the light intensity for photosynthesis (Vaseghi et al., 2018). We provide an MP-based validation of this interaction. We find that addition of reduced TRX, leads to an increase in the decamer abundance, a sign of reduction of 2CP (Figure 2B). Complementary approaches with intact plants, *in vitro* biochemistry and mathematical modeling validated this function (Vaseghi et al., 2018; Telman et al., 2020; Gerken et al., 2020). Future models should include the presence of pools of differently efficient thiol peroxidases in order to estimate their contribution to the regulatory cycle. K_M_(H_2_O_2_)-values of bacterial and plant 2CP were reported with 1–2 μM (Poole, 2008; Parsonage et al., 2008). Here, it is intriguing that 100 nM H_2_O_2_ was able to oxidize a major portion of 2CP confirming the very high substrate affinity of 2CP and inactivation by hyperoxidation after approximately 250 catalytic cycles (König et al., 2013).

The function of hyperoxidized 2CP as a chaperone may be significant but hardly explains the physiological advantage of the delicate dimer-oligomer-multimer equilibria of the reduced form with far less chaperone activity (Jang et al., 2004; König et al., 2003). Detailed studies in *Arabidopsis*, barley and potato revealed species- and isoform-specific variation in the susceptibility of 2CP to hyperoxidation (Cerveau et al., 2019). Specific interactions of 2CP with other proteins such as β-carbonic anhydrase may explain the conserved redox-dependent conformational dynamics (Liebthal et al., 2020).

The comparison of the oligomeric distributions of 2CP, HsPRX1, HsPRX2 and HsPRX3 revealed distinct distribution patterns of dimers, decamers, eicosamers and eicosamer stacks. We found considerable similarity between 2CP and HsPRX1 suggesting homologous functions as discussed above. A significant fraction of recombinant HsPRX1 adopted the decameric conformation at low concentration (Figure 4). Notably, human PRXs undergo profound posttranslational modifications such as phosphorylation, glutathionylation and acetylation, pointing toward future MP-based studies of the effects of these modifications on oligomerization propensity and thus redox function (Rhee and Woo, 2020).

The cytosolic HsPRX1 and HsPRX2 proteins function e.g., in tumor suppression and red blood cell maintenance without functional redundancy (Chang et al., 2004). This colocalization of highly similar PRXs indicates functional differences possibly linked to oligomer distribution. This may alter their reactivity with ROS or interaction with partner proteins and shape their physiological function, e.g., in cardiovascular and neurological disorder, carcinogenesis or apoptosis (Yewdall et al., 2016; Kim and Jang, 2019; Baxter and Hardingham, 2016). The current understanding of PRX function suggests that PRX-dependent signaling involves formation of transient or stable complexes between target proteins, scaffold proteins and PRXs. In the case of the regulation of the human transcription factor STAT3, which is oxidized by HsPRX2, the interaction is stabilized by the presence of ANNEXIN A2 (Talwar et al., 2020). Furthermore, HsPRX3 forms stacks and tubes depending on pH and redox state (Ardini et al., 2021). Reduced HMW aggregates of HsPRX3, as shown here, may serve as storage pools to release dimers and decamers for efficient peroxide detoxification controlling apoptosis.

### Limitations of the study

The study provides a methodological blueprint for dissecting the relationship between particular amino acid residues, oligomerization state and the function of 2-Cys peroxiredoxins. At present, the limited number of explored site directed mutagenized variants defines some extreme cases, but a detailed comparison of the primary to quaternary structures of the four 2-Cys peroxiredoxins explored here and the inclusion of additional ones will allow for identifying additional decisive amino acid residues which define the unique differences in transition dynamics. In addition, it will be important to introduce interaction partners into the system in order to understand their effect on the transition dynamics and ultimately *in vivo* function.

## STAR★Methods

### Key resources table

**Table:** 

REAGENT or RESOURCE	SOURCE	IDENTIFIER
**Bacterial and virus strains**		

BL21(DE3)	NEW ENGLAND BioLabs Inc.	C2527H

**Recombinant DNA**		

pAt2CPA	Dietz Lab (Bielefeld University)	N/A
pHsPRX1	Dietz Lab (Bielefeld University)	N/A
pHsPRX2	Dietz Lab (Bielefeld University)	N/A
pHsPRX3	Dietz Lab (Bielefeld University)	N/A
pAtTRX-x	Dietz Lab (Bielefeld University)	N/A
pAtTRX-f1	Dietz Lab (Bielefeld University)	N/A
pAtTRX-m2	Dietz Lab (Bielefeld University)	N/A
pAt2CPF84R	Dietz Lab (Bielefeld University)	N/A
pAt2CPC54D	Dietz Lab (Bielefeld University)	N/A
pAt2CPC54S	Dietz Lab (Bielefeld University)	N/A

**Software and algorithms**		

DiscoverMP 2.0.0	Refeyn Ltd.	N/A
Omnisize 3.0	Viscotek Corp.	N/A

**Other**		

OneMP mass photometer	Refeyn Ltd.	N/A
Viscotek 802 DLS	Viscotek Corp.	N/A

### Resource availability

#### Lead contact

• Further information and requests for resources and reagents should be directed to and will be fulfilled by the lead contact, Philipp Kukura (Philipp.kukura@chem.ox.ac.uk).

#### Materials availability

• This study did not generate new unique reagents.

### Experimental model and subject details

#### Bacterial strains

Recombinant proteins were overexpressed in *E. coli* BL21(DE3) strain using Lysogeny Broth (LB) medium. The cells were grown at 37°C until OD_600_ reached 0.8 and then induced with 0.4 mM Isopropyl b-D-1-thiogalactopyranoside (IPTG). After 4 h at 37°C the cells were harvested.

#### Recombinant proteins expression and purification

Bacterial expression vectors of wildtype and variants of At2CPA (F84R, C54D, C54S) were generated in a previous study (König et al., 2013). All recombinant proteins were expressed, purified, and treated according to Liebthal et al. (2016). Dr. Thorsten Seidel (Bielefeld University) supplied the plasmids for expression of human HsPRX1, HsPRX2 and HsPRX3 (Barranco-Medina et al., 2008). The recombinant proteins were reduced after purification by Ni-NTA-affinity chromatography with 10 mM DTT for 1 h. Samples were dialyzed 3 times for 4 h each in order to change the buffer and remove DTT and imidazole (35 mM HEPES, pH 8, 100 mM NaCl). Aliquots were snap-frozen in liquid nitrogen, stored at −80°C, and immediately thawed before each experiment. All proteins were prepared 20 min prior analysis. Recombinant TRXs (TRX-x, TRX-f1, TRX-m1) were expressed, prepared and treated as described in Vaseghi et al. (2018).

#### Dynamic light scattering

All protein samples were reduced and analyzed at 10 μM concentration in a Viscotek 802 DLS with the OmniSIZE 3.0 DLS software. The mass distribution spectrum of each protein was determined in 10 s time frames with 10 iterations at 20°C and 50% laser power in a 90° measurement angle. Data filtering was performed with 20% spike tolerance and 15% baseline drift. Distributions were calculated according to the protein mass model.

### Quantification and statistical analysis

#### Mass photometry

All proteins were reduced as described above and diluted with fresh and degassed buffer (35 mM HEPES, pH 8, 100 mM NaCl) if not stated otherwise. At least two different batches of purified protein were used for each study. A standard protein solution was daily used for calibrating the contrast intensity to mass values. MP data was acquired with 5-fold frame averaging below 1 μM protein concentration and with 2-fold frame averaging at 1 μM concentration and higher using a OneMP mass photometer (Refeyn Ltd). The resulting video data was analyzed using DiscoverMP software (Refeyn, Oxford, UK). Raw contrast values were converted to molecular mass using a standard mass calibration, and binding events combined in 5 kDa bin width. Binding events below 40 kDa were indistinguishable from background. Detection settings were adjusted according to the specific visualization requirements and with a background reading of buffer alone. Quantification of oligomer populations was achieved by integrating the peaks around the assumed size of the respective oligomers and by calculation according to the total binding events or 2CP population. Statistics were obtained from n > 6 readings using two independently expressed and purified protein samples and measurements on at least two different days. Statistical parameters as well as the number of independent repeats for individual datasets are referred at the relevant figures.

## Data Availability

• Raw video data reported in this paper will be shared by the lead contact upon request. • This paper does not report original code. • Any additional information required to reanalyze the data reported in this paper is available from the lead contact upon request.
