# Supplementary material for: Single molecule mass photometry reveals the dynamic oligomerization of human and plant peroxiredoxins
Source: iScience. 2021 Oct 13;24(11):103258. doi: 10.1016/j.isci.2021.103258 (PMC8571717; doi:10.1016/j.isci.2021.103258)
Supplement: Document S1. Figures S1–S6 [file mmc1.pdf]

iScience, Volume *24*

Supplemental information

Single molecule mass photometry reveals the dynamic oligomerization of human and plant peroxiredoxins

Michael Liebthal, Manish Singh Kushwah, Philipp Kukura, and Karl-Josef Dietz

Supplementary Figures:

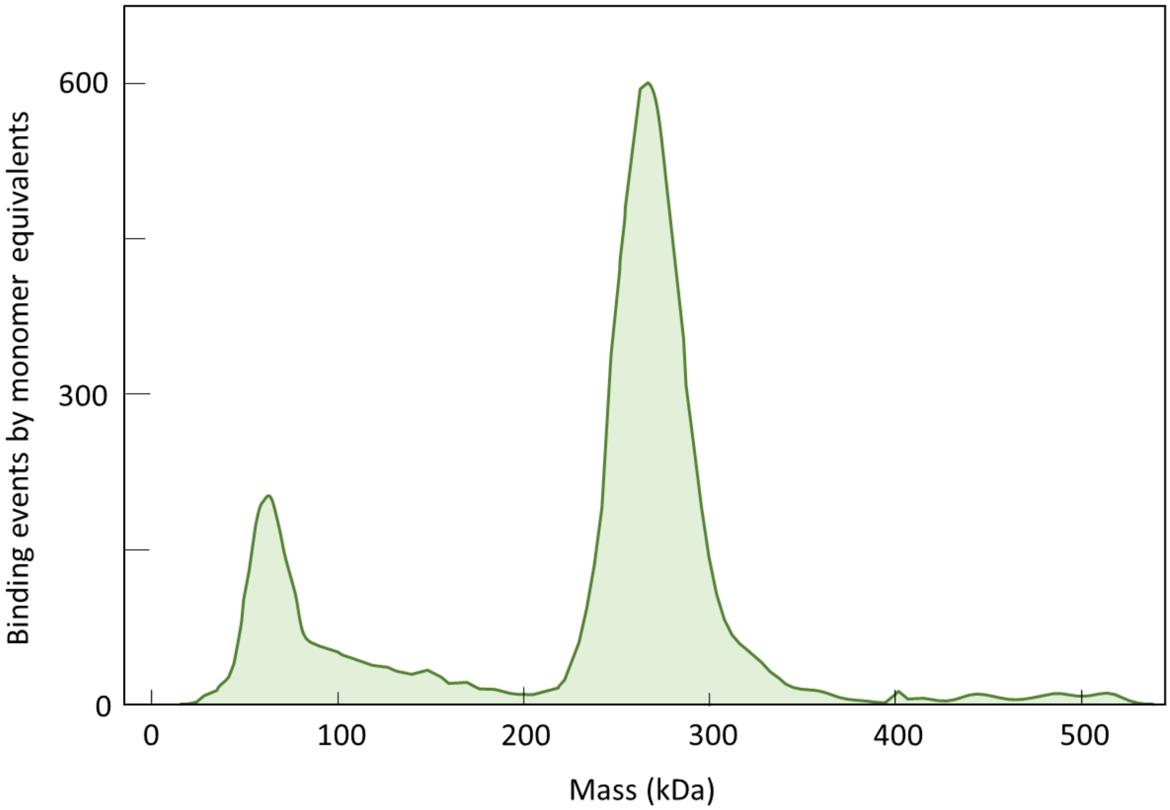

Supplementary Figure 1. Oligomer distribution of plant At2CPA at 100 nM concentration plotted as monomer equivalents, Related to Figure 1. Prior to analysis, the protein was reduced with DTT and diluted with degassed buffer (35 mM HEPES, pH 8, 100 mM NaCl). Recordings were taken after 20 min to exclude unequal dilution or oligomerization dynamics. Similar results were seen in n>12 experiments. The plot was generated based on averaged values similar as presented in **Supplementary Figure 2.**

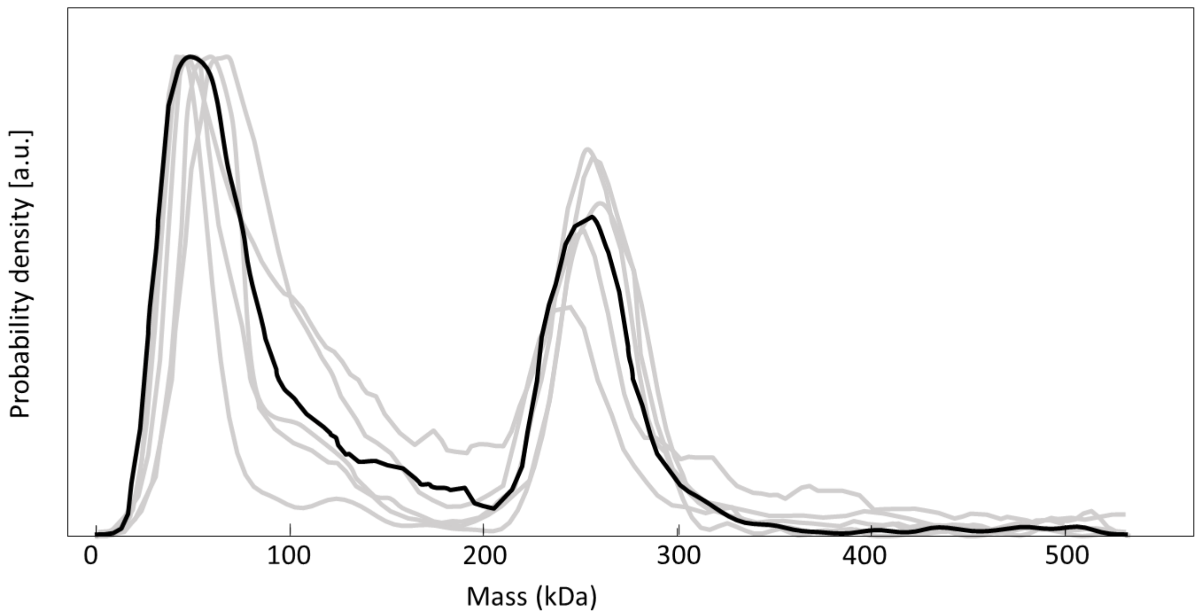

Supplementary Figure 2. Reproducibility of oligomer distribution determined by mass photometry, Related to Figure 1. The At2CPA proteins were reduced with DTT and diluted with degassed buffer (35 mM HEPES, pH 8, 100 mM NaCl). Recordings were performed 20 min later to exclude unequal dilution or oligomerization dynamics. The overlay for 50 nM 2CPA consists of n=5 independent readings (grey) and the averaged result (black) which is used in all figures.

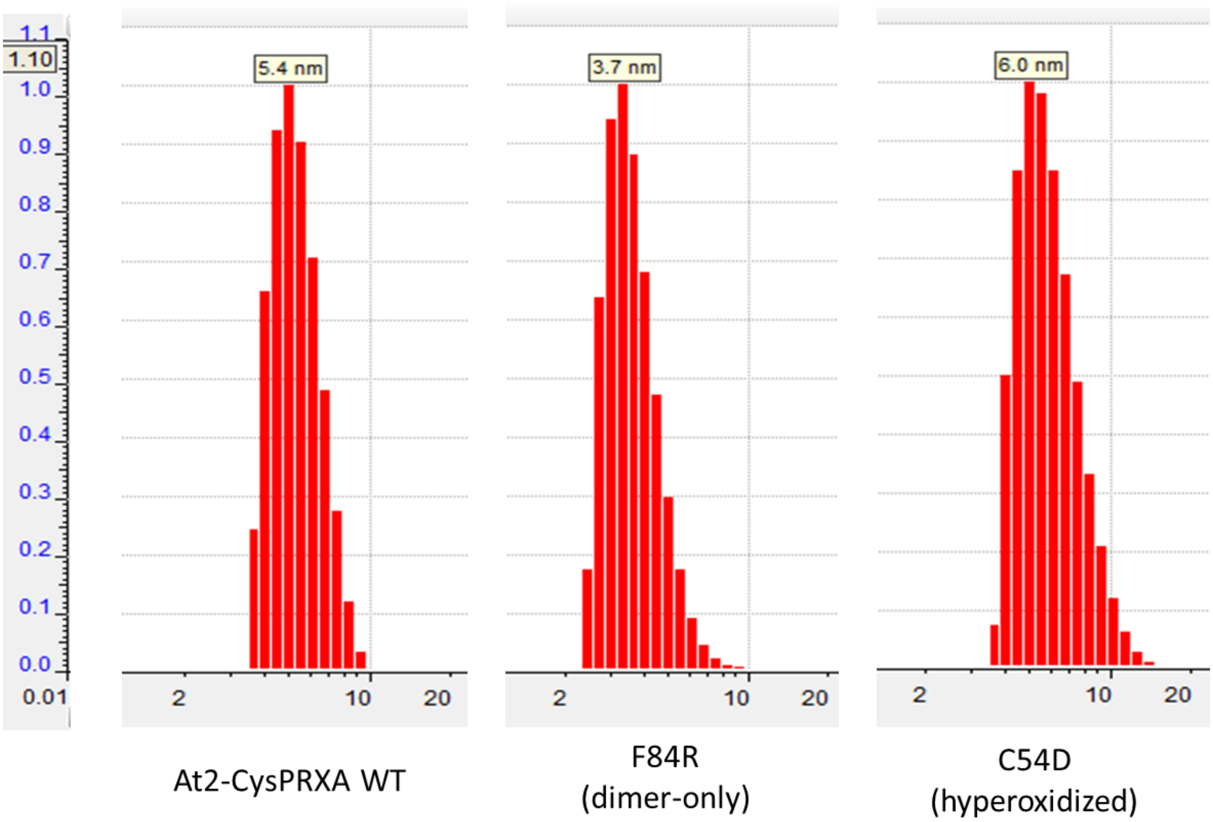

Supplementary Figure 3. Dynamic light scattering of At2CPA using 10 µM wildtype, F84R, and C54D, Related to Figure 1. The samples were prepared as described for mass photometry and analyzed using a Viscotek 802 DLS with the OmniSIZE 3.0 DLS software. Recordings were performed 20 min later to exclude unequal dilution or oligomerization dynamics. The dominant peak corresponding to the respective protein size (25 – 480 kDa) is depicted while other peaks describing impurities are excluded.

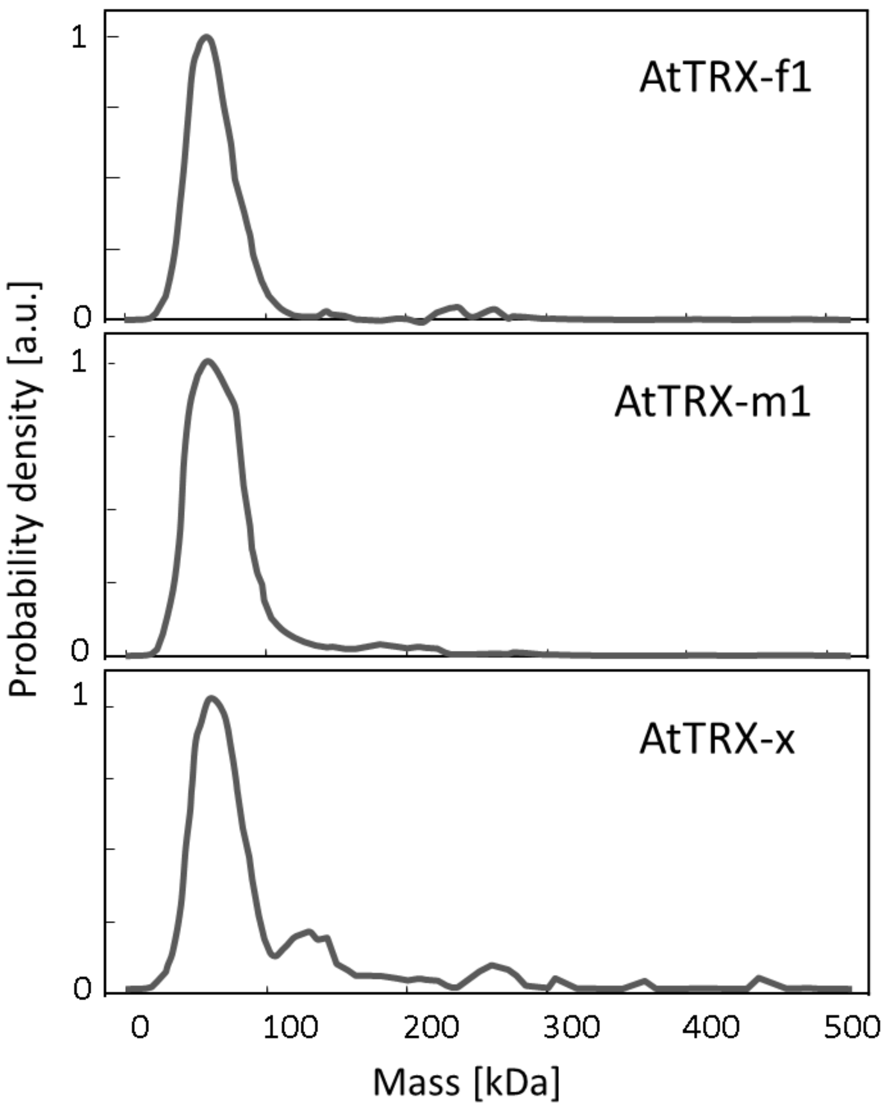

Supplementary Figure 4. Mass photometry readings of plant thioredoxins (TRX-f1/ TRX-m1/ TRX-x), Related to Figure 2. Mass photometry readings of plant thioredoxins (TRX-f1/ TRX-m1/ TRX-x). The proteins were reduced by DTT, diluted with degassed buffer (35 mM HEPES, pH 8, 100 mM NaCl) and analyzed with mass photometry at 100 nM. Recordings were taken after 20 min. Similar results were seen in n=3 experiments.

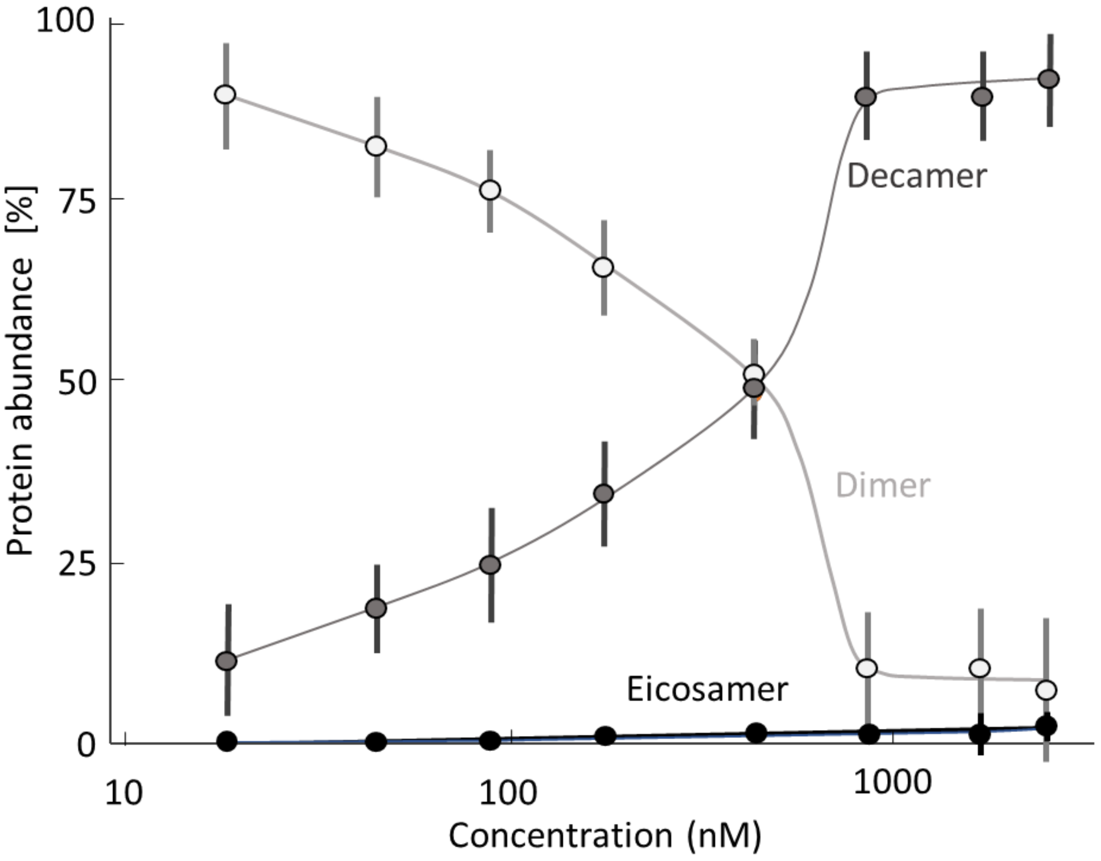

Supplementary Figure 5. Relative abundance of oligomers of HsPRX1 as a function of concentration ranging from 20 nM to 3 µM, Related to Figure 4. Proteins were reduced by DTT, diluted with degassed buffer (35 mM HEPES, pH 8, 100 mM NaCl) and analyzed with mass photometry. Recordings were taken after 20 min. The plots were generated based on averaged values similar as presented in **Supplementary Figure 2**. To estimate the actual share in each distribution, the areas for dimers (0 to 100 kDa), decamers (200 to 300 kDa) and eisosamers (400 to 600 kDa) were integrated and compared to the total distribution area.

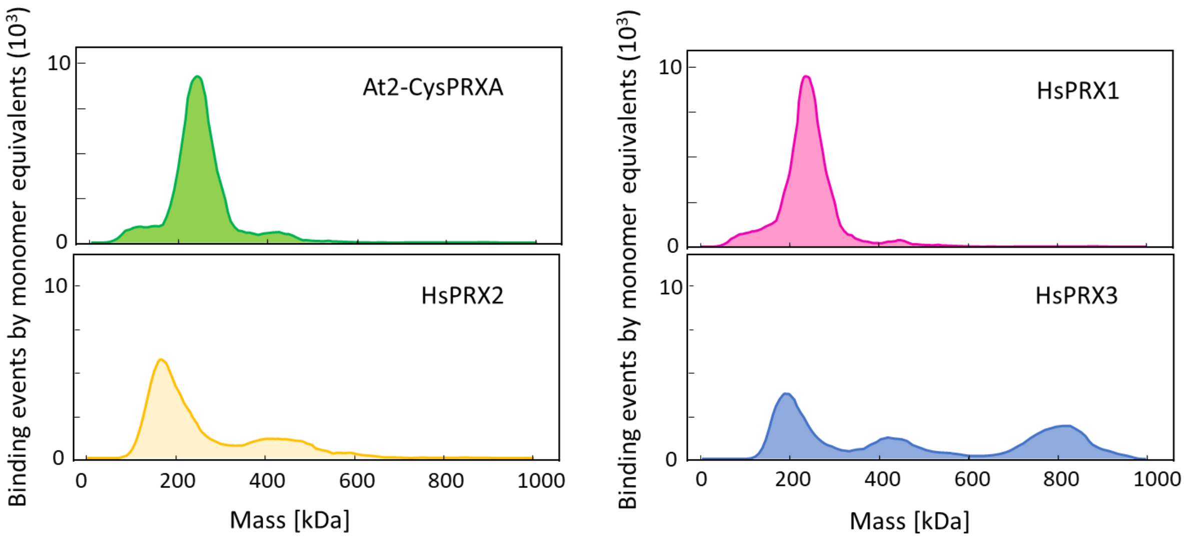

Supplementary Figure 6. Oligomer distribution of plant and human 2CP at 2 µM monomer concentration., Related to Figure 4. Recombinant proteins were analyzed by mass photometry at a concentration of 2 µM. Prior to analysis, the protein was reduced with DTT and diluted with degassed buffer (35 mM HEPES, pH 8, 100 mM NaCl). Measurements were taken after 20 min. Similar results were seen in n>8 experiments.
